# Frequency of Hyponatremia in Patients of Tuberculosis Bacterial Meningitis in a Tertiary Care Hospital

**DOI:** 10.7759/cureus.13888

**Published:** 2021-03-14

**Authors:** Ameet Kumar, Jay Singh, Owais Hashmat, Parma Ameet, Neeraj Budhrani, Khalid Sher

**Affiliations:** 1 Department of Neurology, Jinnah Postgraduate Medical Centre, Karachi, PAK; 2 Department of Neurology, Dow University of Health Sciences, Karachi, PAK; 3 Department of Neurology, The Aga Khan University, Karachi, PAK

**Keywords:** tuberculous bacterial meningitis, hyponatremia, cerebral salt wasting syndrome, syndrome of inappropriate antidiuretic hormone

## Abstract

Introduction

Tuberculous meningitis (TBM) brings significant morbidity and mortality worldwide. Hyponatremia has long been documented as a potentially grave metabolic result of TBM. The syndrome of inappropriate antidiuretic hormone (SIADH) secretion has been supposed to be accountable for the majority of cases of hyponatremia in TBM. Cerebral salt wasting syndrome (CSWS) is being progressively reported as a basis of hyponatremia in some of these cases. Differentiating CSWS from SIADH can be challenging but is vital because treatment of these two conditions is profoundly different.

Objective

The rationale of our study is to determine the frequency of hyponatremia and etiology in patients presenting with TBM in a tertiary care hospital in order to establish the local perspective as there is paucity of local data.

Methods

A total of 160 hospitalized patients at a tertiary care hospital in Pakistan who fulfilled the inclusion criteria were enrolled in this study after informed consent. The study was conducted for six months at the department of neurology, Jinnah Postgraduate Medical Centre (JPMC), Karachi, Pakistan. Brief history was taken and demographic information was entered in the performa by researchers. The data was collected and analyzed on Statistical Package for Social Sciences (SPSS) version 18.0 (IBM Corp., Armonk NY, USA). Demographic data were presented as simple descriptive statistics giving mean and standard deviation for age, height, weight, GCS (Glasgow Coma Scale), serum sodium and duration of symptoms. Frequencies and percentages were calculated for categorical variables like gender, hypertension, smoking status, T2DM (Type 2 Diabetes Mellitus), BMRC (British Medical Research Council Contemporary Clinical Criteria for TBM) stage, hyponatremia, SIADH and CSWS. Effect modifiers were controlled through stratification of age, gender, hypertension, smoking status, T2DM, BMRC stage and duration of symptoms to see the effect of these on the outcome variable (hyponatremia). Quantitative data were presented as simple descriptive statistics giving mean and standard deviation and qualitative variables were presented as frequency and percentages. Post stratification chi-square test was applied with a p-value of ≤0.05 taken as significant.

Results

In our study, out of 160 patients with TBM, 40% (64) had hyponatremia. Moreover, 14.4% and 25.6% had SIADH and CSWS, respectively with 60% (96) of patients were male and 40% (64) were female. Mean age of patients in our study was 46.78±2.81 years. Whereas, mean duration of symptoms, serum sodium, GCS, height and weight in our study was 1.2±0.78 weeks, 128.65±7.52 mmol/L and 11.21±3.14%, 158±7.28 cm and 78.7±9.87 kg, respectively.

Conclusion

This study concluded that the frequency of hyponatremia among patients of TBM was significant, consistent with previous studies. Privation of proper assessment and management can lead to grave and permanent neurological consequences, as well as death. Healthcare providers should be aware of the implication of sodium deregulation among patients of TBM and differentiate between the numerous therapeutic preferences in order to advocate safe and effective treatment.

## Introduction

Tuberculosis (TB) still remains one of the foremost infectious sources of morbidity and mortality globally [1]. According to the World Health Organization (WHO) global TB report 2013, there were 8.6 million incident cases of TB with an expected mortality of 1.3 million [2]. Central nervous system (CNS) tuberculosis is the third most common manifestation of extra-pulmonary tuberculosis as supported in a local study [3]. Among CNS tuberculosis, tuberculous meningitis (TBM) is the commonest presentation with high morbidity and mortality [4]. Hyponatremia is the most common electrolyte irregularity observed in admitted patients of TBM [5]. Sodium disorders are related with considerable morbidity and mortality. Due to high prevalence and potential neurological impediments, it is mandatory to consider differential diagnosis of hyponatremia, before any therapeutic intervention [6].

Hyponatremia is a common electrolyte disorder and is a common outcome in patients with acute cerebral insult [7]. Hyponatremia in CNS infection can be result of more than single mechanism, syndrome of inappropriate antidiuretic hormone (SIADH) secretion being the foremost mechanism. The other mechanism is noted to be cerebral salt wasting syndrome (CSWS) [8]. As described, SIADH is a volume-expanded state because of antidiuretic hormone-facilitated renal water holding [9]. CSWS is described by a constricted effective arterial blood volume subsequent of renal salt wasting [10]. The precise mechanism essential for cerebral salt wasting syndrome remains imprecise. Natriuretic peptides, including both brain natriuretic peptide and C-type natriuretic peptide, have been long associated with the proposed mechanism [11]. The pathogenesis of SIADH is linked to the damage of the hypothalamic cells affecting leakage of ADH into the circulation [12]. Another mechanism is believed to be involving resetting of the osmoreceptors located in the hypothalamus [13]. It is vital for a clinician to be able to differentiate between SIADH and CSWS logically as they both present with the same phenomenon of hyponatremia. It is also crucial as these two conditions develop under comparable settings [14]. It is worth-mentioning that the treatment of each of it is different warranting precise diagnosis by clinicians. On one hand, SIADH is treated with fluid restriction. On the other hand, CSWS is treated with fluids and improvement of hyponatremia [15]. Misra, et al. assessed cases of TBM and established the prevalence of hyponatremia to be 44.7%; and amongst the patients with hyponatremia 50% and 8.82% were linked to CSWS and SIADH, respectively [16].

## Materials and methods

We conducted a six-month cross-sectional study of consenting patients admitted with a diagnosis of TBM at the Department of Neurology, Jinnah Postgraduate Medical Center (JPMC), Karachi, Pakistan. In our study, inclusion criteria included all patients of either sex aged 18 to 60 years. Non-consenting patients were excluded from the study along with those patients with history of mania, bipolar effective disorder or posttraumatic stress, lung carcinoma, hypothyroidism or hyperthyroidism, Addison’s disease, tuberculosis, head trauma, multiple sclerosis, asthma, renal impairment, congestive heart failure, myocardial infarction, chronic obstructive pulmonary disease and chronic liver disease. A brief history of the duration of illness and demographic information was taken from each patient and confirmed by an attendant. Blood samples were drawn by the researchers and sent for the measurement of serum urea, electrolytes, thyroid function tests, short synacthen test and urinary sodium at the time of admission and transported to hospital standardized laboratory by proper labeling as well as the investigation requested. Patients were labeled as having hyponatremia, SIADH and CSWS as per operational definitions. The findings were noted in the performa along with other demographic and confounding variables like hypertension, smoking status, T2DM (type 2 diabetes mellitus), BMRC (British Medical Research Council Contemporary Clinical Criteria for TBM) stage I/II/III and duration of symptoms.

Patients were diagnosed with TBM if presenting with any two or more of the clinical features in one week: Fever ≥ 99°F occurring about at least 6 hours/day for more than one week, headache dull in nature persisting on VAS (visual analog scale) ≥ 6 for at least three hours/day per day for more than one week with 0 as no pain and 10 being most severe pain, vomiting of any amount at least three times per day for three consecutive days or history of contact with TB patient in family (living in the same house) or outside (outside the house) in last two years. Apart from clinical features presence of any one of the positive laboratory data was used to label TBM: positive acid-fast bacilli (AFB) smear on cerebrospinal fluid (CSF), positive AFB culture on CSF, CSF pleocytosis (20-500 lymphocytes per cubic mm) with increased CSF protein ≥ 100 mg/dl and decreased CSF glucose concentration <60% of corresponding plasma level checked at the same time as CSF examined. Patients having serum sodium level ≤135 mEq/L were labeled as having hyponatremia. Patients with hyponatremia was evaluated for the etiology of hyponatremia and patients was labeled as having SIADH or CSWS based on the volume status; Euvolemia (absence of supine heart rate more than 100 and systolic BP less than 100 mmHg) was labeled SIADH and hypovolemia (presence of supine heart rate more than 100 and systolic BP less than 100 mm Hg) was labeled as CSWS, plus having any two or more of the following: Plasma sodium concentration ≤135 mmol/L, Plasma osmolality ≤280 mOsmol/kg, Urine osmolality ≥100 mOsmol/kg, Urinary sodium concentration ≥30mmol/L, absence of clinical or biochemical features of adrenal and thyroid dysfunction or no history of diuretic use within the past three months. Patients were labeled as hypertensive if known hypertensive for more than two years on treatment and compliant (patients taking medicines regularly and having SBP ≤ 140 mmHg and ≤ 90 mmHg for more than six months). Patients were labeled as diabetic if known diabetic on treatment for more than two years on treatment and compliant (patients taking medicines regularly (daily) or having HbA1C ≥ 6.5%. Patient was labeled as smoker if a person smokes at least five cigarettes a day for at least one year.

The data were collected and analyzed on Statistical Package for Social Sciences (SPSS) version 18.0 (IBM Corp., Armonk NY, USA). Demographic data were presented as simple descriptive statistics giving mean and standard deviation for age, height, weight, GCS (Glasgow Coma Scale), serum sodium and duration of symptoms. Frequencies and percentages were calculated for categorical variables like gender, hypertension, smoking status, DM, BMRC stage, hyponatremia, SIADH and CSWS. Effect modifiers were controlled through stratification of age, gender, hypertension, smoking status, DM, BMRC (British Medical Research Council Contemporary Clinical Criteria for TBM) stage and duration of symptoms to see the effect of these on the outcome variable (hyponatremia). Quantitative data were presented as simple descriptive statistics giving mean and standard deviation and qualitative variables was presented as frequency and percentages. Post-stratification chi-square test was applied with a p-value of ≤0.05 taken as significant. We applied non-probability consecutive sampling. For sample size calculation we used OpenEpi, by taking frequency of SIADH 8.82% [16], margin of error= 5% and confidence level ‘CI’=95%; the required sample size came out to be 160.

## Results

A total of 160 patients diagnosed with TBM were included in this study according to the inclusion criteria. Out of 160 patients with TBM, 40% (64) had hyponatremia. It was also found that 14.4% (23) had SIADH and 25.6% (41) had CSWS. The age range of the patients was 26 years being minimum while the maximum age of the patients was 60 years. Mean age in our study was 46.78 years with a standard deviation of ±2.81. Whereas, mean duration of symptoms, serum sodium, GCS, height and weight in our study was 1.2±0.78 weeks, 128.65±7.52 mmol/L (range: 125-135) and 11.21±3.14, 158±7.28 cm and 78.7±9.87 kg respectively. In terms of gender distribution among cases of our study, 60% (96) of our participants were male and 40% (64) were female.

**Figure 1:**
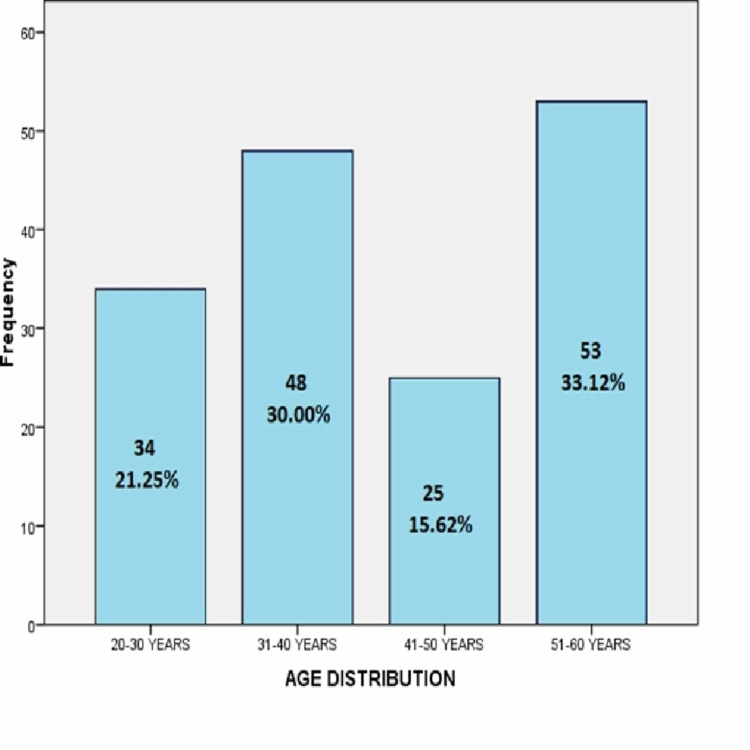
Age distribution (n=160).

Frequency distribution of age showed that out of 160 patients with TBM, 21.2% (34), 30% (48), 15.6% (25) and 33.1% (53) patients were in the age group 20-30 years, 31-40 years, 41-50 years and 51-60 years respectively as presented in Figure 1.

**Figure 2:**
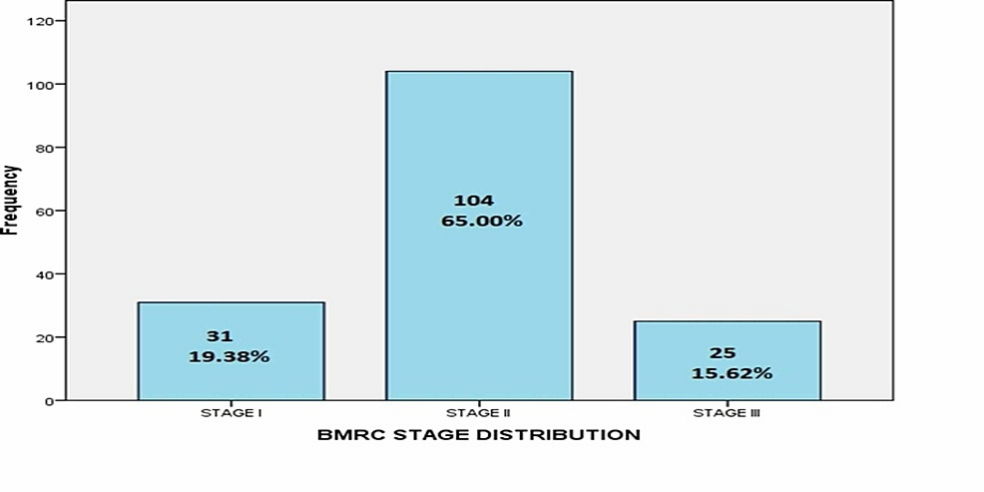
BMRC stage distribution (n=160). BMRC: British Medical Research Council.

Frequency distribution of duration of symptoms showed that out of 160 patients with TBM, 50.6% (81) and 49.4% (79) had symptoms for less than or greater than one week respectively. Frequency distribution of BMRC (British Medical Research Council) stage for TBM showed that out of 160 patients with TBM, 19.4% (31), 65% (104) and 15.6% (25) patients were in BMRC stage I, II and III, respectively, as presented in Figure 2.

In our study, 23 (14.4%) have T2DM. It was also noted in our study that 17 (10.6%) had hypertension and 26 (16.2%) were smokers.

Stratification for age with respect to hyponatremia showed that 16 (25%), 23 (35.9%), 06 (9.4%) and 19 (29.7%) patients who were in the age group 20-30 years, 30-40 years, 41-50 years and 51-60 years had hyponatremia, respectively. Whereas 18 (18.8%), 25 (26%), 19 (19.8%) and 34 (35.4%) patients who were in the age group 20-30 years, 31-40 years, 41-50 years and 51- 60 years did not have hyponatremia, respectively (Tables 1, 2).

**Table 1:** Descriptive statistics (n=160). GCS: Glasgow Coma Scale.

Variables	Mean±SD	Min-Max
Age (years)	46.78±2.81	26-60
Duration of symptoms (weeks)	1.2±0.78	0.2-1.8
Serum sodium (mmol/L)	128.65±7.52	125-135
GCS	11.21±3.14	7-14
Height (cm)	158±7.28	148-162
Weight (kg)	78.7±9.87	68-115

**Table 2:** Hyponatremia according to age (n=160).

Age (years)	Hyponatremia		Total
	Yes	No	
20-30	16 (25%)	18 (18.8%)	34 (21.2%)
31-40	23 (35.9%)	25 (26%)	48 (30%)
41-50	06 (9.4%)	19 (19.8%)	25 (15.6%)
51-60	19 (29.7%)	34 (35.4%)	53 (33.1%)
Total	64 (100%)	96 (100%)	160 (100%)
p-value	0.17		

Stratification for gender with respect to hyponatremia showed that 34 (53.1%) and 62 (64.6%) who were in the male group had and did not have hyponatremia respectively. Whereas 30 (46.9%) and 34 (35.4%) who were in the female group had and did not have hyponatremia, respectively. p-value was 0.10 as presented in Table 3.

**Table 3:** Hyponatremia according to gender (n=160).

Gender	Hyponatremia		Total
	Yes	No	
Male	34 (53.1%)	62 (64.6%)	96 (60%)
Female	30 (46.9%)	34 (35.4%)	64 (40%)
Total	64 (100%)	96 (100%)	160 (100%)
p-value	0.10		

Stratification for the duration of symptoms with respect to hyponatremia showed that patients who had symptoms < 1 week, 35 (54.7%) had hyponatremia. Whereas patients who had symptoms > 1 week, 29 (45.3%) had hyponatremia. p-value was 0.24 as presented in Table 4.

**Table 4:** Hyponatremia according to the duration of symptom status (n=160).

Duration of symptoms	Hyponatremia		Total
	Yes	No	
< 1 week	35 (54.7%)	46 (47.9%)	81 (50.6%)
> 1 week	29 (45.3%)	50 (52.1%)	79 (49.4%)
Total	64 (100%)	96 (100%)	160 (100%)
p-value	0.24		

Stratification for BMRC stage with respect to hyponatremia showed that patients who had BMRC stage I, 10 (15.6%) had hyponatremia. Whereas patients who had BMRC stage II & III, 43 (67.2%) and 11 (17.2%) had hyponatremia, respectively. p-value was 0.60 as presented in Table 5.

**Table 5:** Hyponatremia according to BMRC stage (n=160) BMRC: British Medical Research Council Contemporary Clinical Criteria for TBM.

BMRC stage	Hyponatremia		Total
	Yes	No	
Stage I	10 (15.6%)	21 (21.9%)	31 (19.4%)
Stage II	43 (67.2%)	61 (63.5%)	104 (65%)
Stage III	11 (17.2%)	14 (14.6%)	25 (15.6%)
Total	64 (100%)	96 (100%)	160 (100%)
p-value	0.60		

Stratification for diabetes mellitus type II with respect to hyponatremia showed that patients who had diabetes mellitus, 07 (10.9%) had hyponatremia. Whereas patients who did not have diabetes mellitus, 57 (89.1%) and 80 (83.3%) had and did not have hyponatremia respectively. p-value was 0.21 as presented in Table 6.

**Table 6:** Hyponatremia according to diabetes mellitus type II (n=160).

Diabetes mellitus	Hyponatremia		Total
	Yes	No	
Yes	07 (10.9%)	16 (16.7%)	23 (14.4%)
No	57 (89.1%)	80 (83.3%)	137 (85.6%)
Total	64 (100%)	96 (100%)	160 (100%)
p-value	0.21		

Stratification for hypertension with respect to hyponatremia showed that patients who had hypertension, 07 (10.9%) had hyponatremia. Whereas patients who did not have hypertension, 57 (89.1%) had hyponatremia. p-value was 0.55 as presented in Table 7.

**Table 7:** Hyponatremia according to hypertension (n=160).

Hypertension	Hyponatremia		Total
	Yes	No	
Yes	07 (10.9%)	10 (10.4%)	17 (10.6%)
No	57 (89.1%)	86 (89.6%)	143 (89.4%)
Total	64 (100%)	96 (100%)	16 (100%)
p-value	0.55		

Stratification for smoking status with respect to hyponatremia showed that patients who smoked, 10.7% (07) had hyponatremia. Whereas patients who did not smoke, 89.1% (57) had hyponatremia and 80.2% (77) did not had hyponatremia. p-value was 0.10 as presented in Table 8. .

**Table 8:** Hyponatremia according to smoking status (n=160).

Smoking status	Hyponatremia		Total
	Yes	No	
Yes	07 (10.9%)	19 (19.8%)	26 (16.2%)
No	57 (89.1%)	77 (80.2%)	134 (83.8%)
Total	64 (100%)	96 (100%)	160 (100%)
p-value	0.10		

## Discussion

In this study, serum sodium levels were evaluated to determine the frequency of hyponatremia in patients presenting with TBM in order to establish the local perspective as there is paucity of local data. A total of 160 patients diagnosed with TBM were included in this study according to the inclusion criteria. Out of 160 patients with TBM, 40% (64) had hyponatremia. It was also found that 14.4% (23) had SIADH and 25.6% (41) had CSWS.

A prospective hospital-based study comprising 76 patients with TBM reported that 34 (44.7%) TBM patients had hyponatremia. It was further observed that hyponatremia was due to cerebral salt wasting in 17, syndrome of inappropriate secretion of antidiuretic hormone in 3 and miscellaneous causes in 14 patients. Outcome of TBM was related to the duration of hospital stay, level of consciousness, abnormal neurological exam, mechanical ventilation, severity of infection, age and other comorbidities [16]. Karandanis, et al. noted in his study that hyponatremia was found in 73% of cases of TBM [17]. Another study noted that mortality was 10% with male population predominantly involved. Hyponatremia in TBM cases was significant (40%). Hydrocephalus, use of mechanical ventilation and TLC more than 9000 were predictors of mortality. After completing anti-tuberculous treatment, 70% of patients had complete recovery without residual deficit while 20% of patients had residual deficit [18].

CNS infections treatment and management is emerging as a great challenge especially in the setting of hyponatremia and its complication. Moreover, differentiating CSWS from SIADH is of paramount importance as both are managed paradoxically but present somewhat similarly. Data from our study would possibly offer new insight to clinicians that can impact the understanding of hyponatremia in TBM and aid in the improvement of active management strategies, improving quality of life and patient well-being.

Our study had a few limitations. Our study used a smaller sample size of admitted patients compared to other studies we discussed. We also used a relatively short duration of study. Future studies with larger populations and longer duration will help mitigate these limitations.

## Conclusions

Our study showed that the prevalence of hyponatremia in patients with TBM is significant, consistent with previous studies. Therefore, as determined in our study, assessment of all patients of TBM is vital for the evaluation of hyponatremia to predict outcome and to improve management. It is also warranted as lack of appropriate evaluation and management can lead to grave and permanent neurological consequences, thereby increasing morbidity and mortality. Clinicians should be aware of the implication of sodium imbalance among patients of TBM and differentiate between the various therapeutic options in order to avoid morbidity and mortality.

## Appendices

BRITISH MEDICAL RESEARCH COUNCIL CONTEMPORARY CLINICAL CRITERIA FOR TBM (BMRC)

• Stage I: Alert and oriented without focal neurological deficits and GCS is 15/15.

• Stage II: Glasgow Coma Score of 11-14 or 15 with focal neurological deficits.

• Stage III: Glasgow Coma Score of 10 or less, with or without focal neurological deficits.
